# Synergistic Anti-Inflammatory Activity of Apolipoprotein A-I and CIGB-258 in Reconstituted High-Density Lipoproteins (rHDL) against Acute Toxicity of Carboxymethyllysine in Zebrafish and Its Embryo

**DOI:** 10.3390/ph17020165

**Published:** 2024-01-28

**Authors:** Kyung-Hyun Cho, Ji-Eun Kim, Dae-Jin Kang, Maria del Carmen Dominguez-Horta, Gillian Martinez-Donato

**Affiliations:** 1 Raydel Research Institute, Medical Innovation Complex, Daegu 41061, Republic of Korea; 2 Center for Genetic Engineering and Biotechnology, Ave 31, e/158 y 190, Playa, La Havana 10600, Cuba

**Keywords:** apolipoprotein A-I (apoA-I), reconstituted high-density lipoproteins (rHDL), CIGB-258, carboxymethyllysine (CML), zebrafish, embryo

## Abstract

CIGB-258 is a 3 kDa altered peptide ligand from heat shock protein (HSP) 60 that exhibits anti-inflammatory activity against the acute toxicity of carboxymethyllysine (CML) with antioxidant and anti-glycation activities via protection of high-density lipoprotein (HDL) and apolipoprotein A-I (apoA-I). It is necessary to test a synergistic interaction between apoA-I and CIGB-258 in reconstituted high-density lipoproteins (rHDL). Several rHDLs were synthesized containing palmitoyloleoyl phosphatidylcholine (POPC), cholesterol, apoA-I, and CIGB-258 at molar ratios of 95:5:1:0, 95:5:1:0.1, 95:5:1:0.5, and 95:5:1:1 for rHDL-(1:0), rHDL-(1:0.1), rHDL-(1:0.5), and rHDL-(1:1), respectively. As the CIGB-258 content in rHDL was increased, the particle size of rHDL was 1.4-times higher than rHDL-(1:0) to rHDL-(1:1), from 60 nm to 83 nm, respectively. As the CIGB-258 content was increased, the rHDL showed the most resistance to isothermal denaturation by a urea treatment, and rHDL-(1:1) exhibited the highest structural stability and the strongest antioxidant ability against LDL oxidation. Co-treatment of rHDL-(1:0), rHDL-(1:0.5), and rHDL-(1:1) resulted in up to 10%, 24%, and 34% inhibition of HDL glycation, inhibition of HDL glycation, which was caused by the CML, with protection of apoA-I. Microinjection of each rHDL into zebrafish embryos in the presence of CML showed that a higher CIGB-258 content in rHDL was associated with higher survivability with the least inflammation and apoptosis. Furthermore, an intraperitoneal injection of rHDL and CML showed that a higher CIGB-258 content in rHDL was also associated with higher survivability of zebrafish and faster recovery of swimming ability. The rHDL-(1:1) group showed the lowest triglyceride, AST, and ALT serum levels with the least production of interleukin-6, oxidized product, and neutrophil infiltration in hepatic tissue. In conclusion, CIGB-258 could bind well to phospholipids and cholesterol to stabilize apoA-I in the rHDL structure against denaturation stress and larger particle sizes. The rHDL containing CIGB-258 enhanced the in vitro antioxidant ability against LDL oxidation, the anti-glycation activity to protect HDL, and the in vivo anti-inflammatory activity against CML toxicity in zebrafish adults and embryos. Overall, incorporating apoA-I and CIGB-258 in rHDL resulted in a synergistic interaction to enhance the structural and functional correlations in a dose-dependent manner of CIGB-258.

## 1. Introduction

Native high-density lipoproteins (HDL) and apolipoproteinA-I (apoA-I) exert antioxidant and anti-inflammatory activities in the blood to suppress proinflammatory responses by inhibiting the oxidation of low-density lipoproteins (LDL) [1,2]. On the other hand, the beneficial functions of HDL can be impaired by oxidative stress, glycation stress, and exogenous infections [3], such as reactive oxygen species (ROS), fructose, and SARS-CoV-2 [4], respectively. A previous study reported that treatment of HDL and apoA-I with fructose or carboxymethyllysine (CML), an advanced glycated end product, elevated proteolytic degradation [4,5,6].

HDL and apoA-I exert cytoprotective and anti-inflammatory activities in endothelial cells by suppressing LDL oxidation and the inflammatory cascade, such as interleukin (IL)-6 production [7,8]. Therefore, enforcement of the structure and functions of apoA-I and HDL are critical to maintaining its anti-inflammatory activity because the quality of HDL can be impaired by exposure to acute infection and oxidative stress to produce dysfunctional HDL [3,9]. CIGB-258 is an altered 3 kDa peptide ligand from heat shock protein (HSP) 60 that consists of 27 amino acids with antioxidant and anti-glycation activities to protect the stability and functionality of HDL and apoA-I [5,6]. The intravenous administration of CIGB-258 (1~2 mg per 12 h) had therapeutic potential to treat hyperinflammation in patients with COVID-19 [10]. These reports imply that HDL and CIGB-258 have anti-inflammatory activity coincidentally by treating COVID-19 via the killing of SARS-CoV-2 by native HDL [4], but the precise interaction between the HDL and CIGB-258 is unclear. 

Nevertheless, there is no information on the putative synergistic activity in the co-presence of apoA-I and CIGB-258 because both proteins and peptides exhibit anti-inflammatory activity. Therefore, it is necessary to determine how CIGB-258 could stabilize the structure of apoA-I and HDL and enhance their functionality to exert synergistic anti-inflammatory activity. A previous study reported that the co-presence of CIGB-258 protected HDL and apoA-I from proteolytic degradation via glycation and oxidation in a dose-dependent manner [5,6]. Furthermore, there is no information on the binding between apoA-I and CIGB-258 regarding a putative protein–peptide interaction to form the HDL structure.

This study synthesized rHDL with human apoA-I (28 kDa) and CIGB-258 at various molar ratios from 1:0.1, 1:0.5, and 1:1 to gain a mechanistic insight into a putative interaction in a lipid-bound state. The rHDLs were characterized and compared regarding the structural change in intrinsic Trp movement in apoA-I, particle morphology, and isothermal denaturation upon increased content of CIGB-258 in rHDL. The movement of an intrinsic fluorophore, especially Trp108, is essential for evaluating the conformational changes in apoA-I, whether enforcement of stability, amyloidogenesis, or proteolytic degradation [11,12,13]. This is because the structural stability of apoA-I is critical to HDL particle formation and HDL functionality, such as antioxidant activity and cholesterol efflux activity, upon binding with serum amyloid A and exchange of apoA-I [14,15]. The functionality of rHDLs was also compared individually regarding the antioxidant ability against LDL oxidation, anti-glycation activity against HDL glycation, and anti-inflammatory activity in zebrafish embryos in the presence of CML, according to previous reports [5,6,14,16]. 

The zebrafish serves as a vertebrate model characterized by well-established innate and acquired immune systems, extensively employed for assessing the toxicity of advanced glycated end (AGE) products [17]. Zebrafish embryos are relatively large-sized and developing externally, offer an excellent model for microinjections into these embryos, facilitating the comparison of developmental morphology and enabling an assessment of the efficacy of a peptide drug. In the current study, the effectiveness of CIGB-258 on enhancing the rHDL stability and functionality, such as antioxidant ability, anti-glycation, and anti-inflammatory activities, were compared with increasing contents of CIGB-258 in the rHDL. 

## 2. Results

### 2.1. Structural Analysis of rHDL Containing ApoA-I and CIGB-258

**Figure 1:**
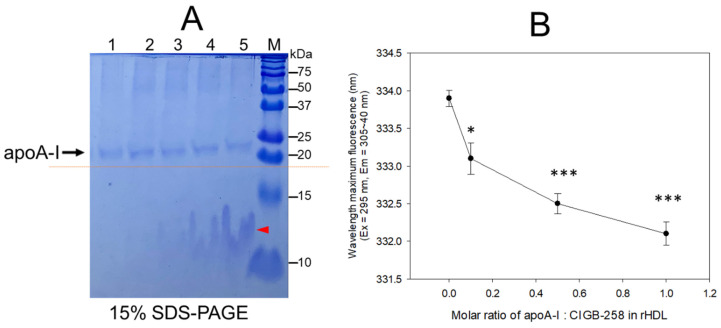
Synthesis of rHDL with apoA-I and CIGB-258 with increasing molar ratio of apoA-I:CIGB-258 from 1:0, 1:0.1, 1:0.5 to 1:1. (**A**) The electrophoretic pattern of rHDL on a 15% SDS-PAGE following Coomassie brilliant blue (0.125%) staining. The dotted red line highlighted the slower electromobility of apoA-I with an increasing molar ratio of CIGB-258. The red arrowhead indicates a mixture of CIGB-258 peptide, cholesterol, and phospholipid. The lanes were assigned as follows: Lane 1, lipid-free apoA-I; Lane 2, rHDL-(1:0); Lane 3, rHDL-(1:0.1); Lane 4, rHDL-(1:0.5); Lane 5, rHDL-(1:1). (**B**) Measurement of wavelength maximum fluorescence (WMF) by fluorospectroscopy at the excitation wavelength (Ex) of 295 nm, and the emission wavelength (Em) 305–400 nm was employed to detect movement of intrinsic Trp in apoA-I with an increase of CIGB-258 content in rHDL. * and *** represent the statistical difference between the groups at *p* < 0.05 and rHDL *p* < 0.001 versus rHDL-(1:0).

An increase in CIGB-258 content in rHDL up to an apoA-I:CIGB-258 molar ratio of 1:0.1, 1:0.5, and 1:1 resulted in the slower electromobility of apoA-I in 15% SDS-PAGE (lane 3–5) with more detection of CIGB-258 peptide and phospholipid debris, as indicated by the red arrowhead in Figure 1A. Upon an increase in CIGB-258 content in rHDL, the wavelength of maximum fluorescence (WMF) of apoA-I decreased from 333.9 nm at a 1:0 molar ratio (rHDL-(1:0) to 332.1 nm at a 1:1 molar ratio (rHDL-(1:1) (Figure 1B). Interestingly, the WMF decreased sharply at rHDL-(1:0.1) around 333.1 nm, while rHDL-(1:0.5) showed 332.5 nm, suggesting that the Trp movement was almost complete by a 1:0.5 molar ratio of apoA-I:CIGB-258. These results suggest that intrinsic fluorophore in apoA-I, most likely Trp108, moved into a more hydrophobic phase as the CIGB-258 content in rHDL was increased in a dose-dependent manner.

### 2.2. Particle Image Analysis

**Figure 2:**
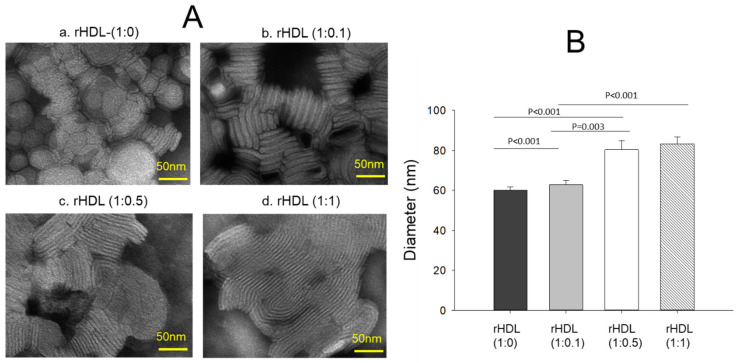
Visualization of the rHDL-containing CIGB-258 particle morphology and diameter through transmission electron microscopy (TEM) (**A**) Morphology analysis at 150,000× magnification, revealing a consistent discoidal shape and rouleaux pattern for all rHDL. (**B**) Mean particle size across the rHDL samples.

Transmission electron microscopy (TEM) revealed an increase in the rHDL particle size as the CIGB-258 content in rHDL was increased in a dose-dependent manner, as shown in Figure 2A. The particle morphology of the rHDL was changed to a more distinct rouleaux pattern, and the particle number also increased as the CIGB-258 content was increased. Measurements of the particle diameter showed that rHDL-(1:0) had the smallest particle diameter of 60 ± 2 nm, while rHDL-(1:0.1), rHDL-(1:0.5), and rHDL-(1:1) had particle sizes of 63 ± 2 nm, 80 ± 4 nm, and 83 ± 3 nm, respectively (Figure 2B). rHDL-(1:1) showed a 1.4-fold larger particle size (photo d, Figure 2A) with a denser rouleaux pattern of particle populations than that of rHDL-(1:0) (photo a). 

### 2.3. Isothermal Denaturation Analysis of rHDL

**Figure 3:**
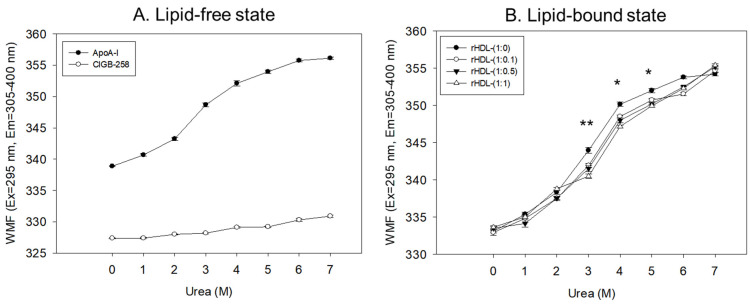
Isothermal denaturation of apoA-I and CIGB-258 in the lipid-free state (**A**) and lipid-bound state (**B**) as reconstituted high-density lipoproteins (rHDL) with different molar ratio of apoA-I: CIGB-258. With increasing urea treatment, change in Trp exposure was compared by fluorospectroscopy (excitation at 295 nm, emission range 305–400 nm) as wavelength maximum fluorescence (WMF). *, *p* < 0.05 between rHDL-(1:0) and rHDL-(1:1); **, *p* < 0.01 between rHDL-(1:0) and rHDL-(1:1).

In a lipid-free state, as shown in Figure 3A, apoA-I and CIGB-258 showed 338 nm and 327 nm of WMF, respectively, in the absence of urea, suggesting that intrinsic fluorophore was remarkably higher in apoA-I because there was no Trp residue in CIGB-258. As the urea treatment was increased, the WMF of apoA-I increased 11 nm to 349 nm at a 3 M urea treatment. Increasing the urea treatment to 7 M resulted in a further 7 nm increase in WMF to 356 nm. Hence, the denaturation of apoA-I increased gradually and sharply until 3 M urea treatment. The denaturation process reached saturation, a typical hyperbolic denaturation pattern of α-helical protein. On the other hand, CIGB-258 showed a linear denaturation pattern with only a 3 nm increase in WMF at 7 M urea without saturation, indicating no α-helical domain. 

In the lipid-bound state, as the urea concentration was increased from 0 M to 7 M, all rHDL exhibited a hyperbolic denaturation process with an increase in WMF from around 332–334 nm to 354–355 nm. Interestingly, rHDL-(1:1) showed the smallest increase in WMF upon the urea treatment, a 2–3 nm blue shift compared to rHDL-(1:0), especially with the 3 M, 4 M, and 5 M urea treatments, suggesting that rHDL-(1:1) was most resistant against urea-induced denaturation (Figure 3B). Interestingly, a higher CIGB-258 content in the rHDL was associated with a smaller extent of denaturation with 3 M–6 M urea, suggesting that CIGB-258 could stabilize more apoA-I via a putative interaction. 

Regression analysis showed that the WMF at median urea treatment (final 3.5 M) decreased as the increase in CIGB-258 content in rHDL increased (Table 1), with a 3.3 nm blue shift of WMF from 347.1 nm (rHDL-0) to 343.8 nm (rHDL-1). By contrast, lipid-free apoA-I showed a WMF of 350.3 nm. Hence, apoA-I in rHDL was more resistant to the denaturation stress of urea than lipid-free apoA-I, showing a 3.2 nm blue shift of the WMF. Furthermore, the incorporation of CIGB-258 induced a larger blue shift of Trp of apoA-I in rHDL in a dose-dependent manner to resist the denaturation stress, implying that the addition of CIGB-258 caused movement of the helix domain in the middle mobile region to stabilize the tertiary structure of apoA-I in the lipid-bound state. As the CIGB-258 content in HDL was increased, intrinsic Trp moved to the non-polar phase via a 3.3 nm blue-shift, and a higher urea concentration was required to reach 50% denaturation (D_1/2_), suggesting a more stabilized secondary and tertiary structure of apoA-I in rHDL by the addition of CIGB-258. 

### 2.4. CIGB-258 Displayed Inhibitory Activity against LDL Oxidation

**Figure 4:**
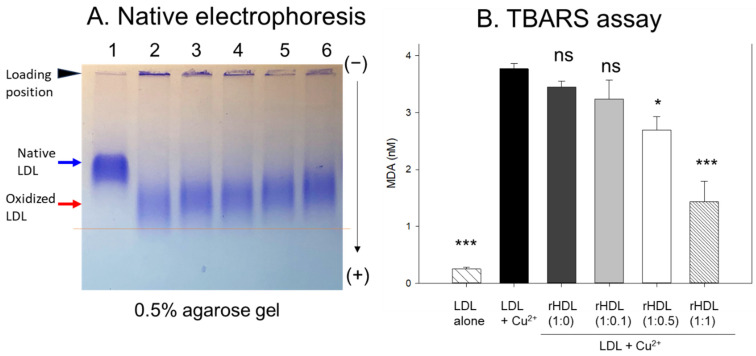
Examining the protective effect of CIGB-258-loaded rHDL against cupric ion-induced oxidative LDL damage. (**A**) 0.5% agarose gel electrophoresis of LDL (10 μg of protein) after exposure to rHDL containing apoA-I and CIGB-258 employing Tris-EDTA buffer (pH 8.0) at 50 V for 1 h following Coomassie brilliant blue staining (1.25%). Lane N represents native LDL. (**B**) Thiobarbituric acid reactive substances (TBARS) quantification in LDL exposed to cupric ions and later subjected to rHDL. The TBARS assays were quantified using malondialdehyde (MDA) as standard. The * and *** express the statistical significance at *p* < 0.05 and *p* < 0.001, respectively, compared to LDL + Cu^2+^ (ox-LDL); ns denoting the non-significant difference compared with ox-LDL.

In agarose gel, native LDL exhibited the strongest intensity and distinct band morphology with the slowest electromobility, as indicated by the blue arrow in Figure 4A (lane 1), whereas oxidized LDL (final 10 μM Cu^2+^ treated) showed a remarkably weaker band intensity with the fastest electromobility and the strongest aggregated band at the loading position, as indicated by the red arrow (lane 2). The more oxidized LDL migrated faster to the bottom of the gel with more diffused and disappeared band intensity because of the apo-B degradation by oxidation, as shown in lane 2, Figure 4A. Conversely, the co-treatment of rHDL-(1:1) in the oxLDL resulted in a stronger band intensity with slower electromobility (lane 6) than the rHDL-(1:0) (lane 3). As the CIGB-258 content in rHDL was increased, the rHDL treatment induced slower electromobility and a less aggregated band in the loading position, as indicated by the black arrowhead, suggesting that the incorporation of CIGB-258 in rHDL induced potent antioxidant activity to inhibit Cu^2+^-mediated LDL oxidation. 

The quantification of oxidation extent through a TBARS assay revealed a 15-fold increase in malondialdehyde (MDA) content in cupric ion-mediated LDL oxidation compared to native LDL (Figure 4B). However, co-treatment with rHDL-(1:1) led to a significant 62% reduction in MDA levels in the oxidized LDL. In contrast, treatment with rHDL-(1:0.1) and rHDL-(1:0.5) resulted in 15% and 29% reductions of MDA, respectively, while treatment with rHDL-(1:0) showed a comparable extent of oxidation to oxLDL alone.

### 2.5. Anti-Glycation Effect of rHDL Containing CIGB-258

**Figure 5:**
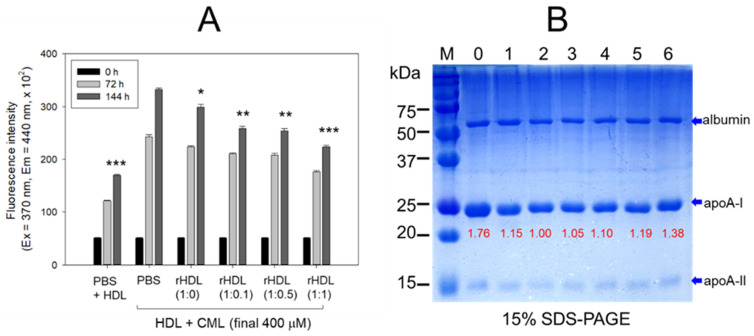
CIGB-258 embedded rHDL exerted anti-glycation efficacy against CML-mediated glycation of HDL. (**A**) Fluorescent intensity was measured at the excitation wavelength (Ex) and emission wavelength (Em) of 370 nm and 440 nm, respectively, in the presence of 400 μM CML after 144 incubation. *, **, and *** express the statistical difference at *p* < 0.05, *p* < 0.01 and *p* < 0.001, respectively, compared to HDL + CML treated group; ns denoting the non-significant difference between the groups. (**B**) HDL’s electrophoretic patterns (15% SDS-PAGE) were analyzed under the influence of 400 μM CML and various rHDL compositions after 144 h incubation. The gel was stained with Coomassie brilliant blue (final 0.125%). The red font highlights the band intensity of apoA-I in three distinct SDS-PAGEs. Lane 0, HDL alone at 0 h; Lane 1, HDL + PBS at 144 h; lane 2, HDL + CML at 144 h; while lanes 3, 4, 5 and 6 represent the HDL + CML with rHDL-(1:0), rHDL-(1:0.1), rHDL-(1:0.5), and rHDL-(1:1), respectively. Lane M, molecular weight marker.

The rHDL containing apoA-I and CIGB-258 displayed potent anti-glycation activity to diminish the yellowish fluorescence in a dose-dependent manner of CIGB-258. Compared with rHDL-(1:0), up to 10%, 24%, and 34% inhibition of HDL glycation were achieved by rHDL-(1:0.1), rHDL-(1:0.5), and rHDL-(1:1), respectively, after 144 h incubation (Figure 5A). Conversely, the co-treatment of rHDL containing CIGB-258 caused a blue shift of the WMF in HDL, especially around 338 nm at rHDL-(1:1), indicating the stabilization of apoA-I in HDL in the presence of CIGB-258 via the inhibition of glycation stress. 

The electrophoresis of the HDL, at 144 h incubation, showed that the treatment of CML into HDL caused remarkable degeneration of the HDL band intensity (lane 2), approximately 15% lower than that of PBS-treated HDL (lane 1), as shown in Figure 5B. Conversely, the co-treatment of rHDL exhibited notable protection against CML-induced proteolytic degeneration of apoA-I in a dose-dependent manner. Specifically, rHDL-(1:0.1), rHDL-(1:0.5), and rHDL-(1:1) demonstrated 10%, 19% and 38% stronger band intensities, respectively. In addition, co-treatment of rHDL-(1:1) resulted in a more distinct band intensity of apoA-I closely resembling apoA-I in native HDL without incubation (lane 0). 

### 2.6. Protection of Zebrafish Embryo against CML-Toxicity

**Figure 6:**
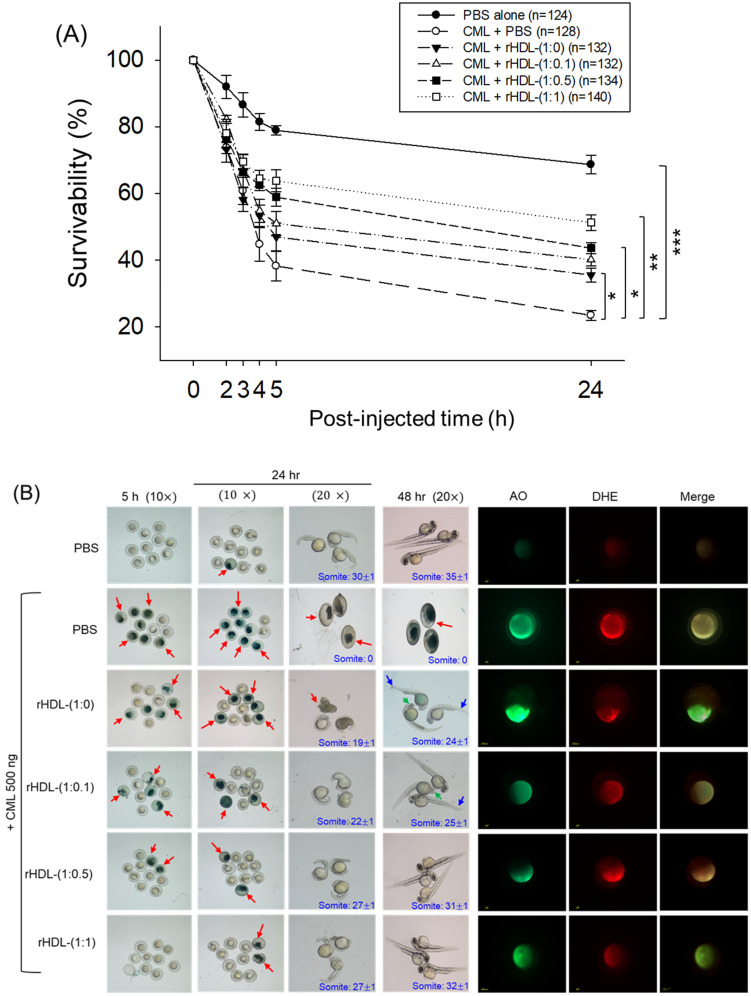

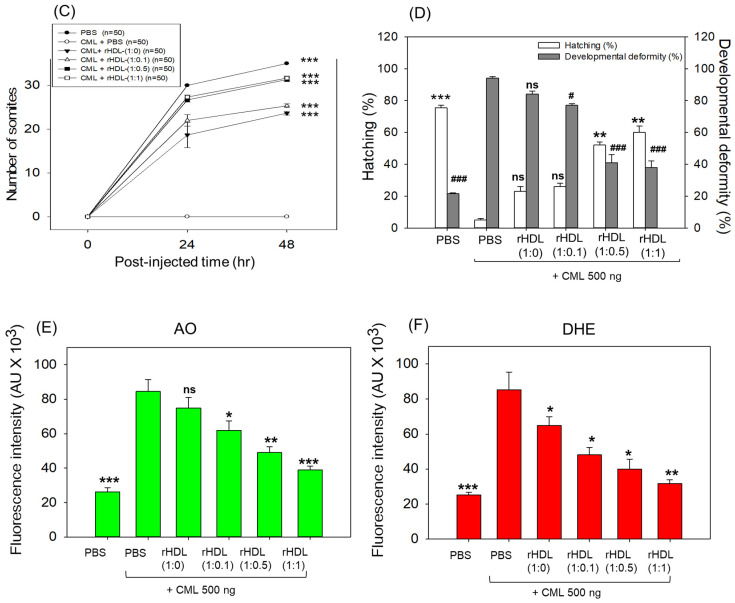
Impact of reconstituted HDL (rHDL) containing CIGB-258 on carboxymethyllysine (CML)-induced toxicity in zebrafish embryos. (**A**) Assessment of zebrafish embryo survivability following exposure to CML and various rHDL treatments over a 72 h post-treatment. *, **, and *** express the statistical difference at *p* < 0.05, *p* < 0.01 and *p* < 0.001, respectively, compared to the CML + PBS group. The ns denoting the non-significant difference between the groups. (**B**) Visual representation of embryos and developmental deformities at 5 h, 24 h and 48 h post-treatment (red arrow indicates the dead embryos, while blue and green arrow denotes the tail fin curvature and reduced eye pigmentation). Dihydroethidium (DHE) and acridine orange (AO) staining represent the production of reactive oxygen species (ROS) and the extent of apoptosis in the embryos at 5 h post-treatment. (**C**) Number of the somites across the different groups during 48 h post-treatment. *** express the statistical difference between the groups at *p* < 0.001, compared to CML + PBS. (**D**) Percentage embryo hatching and developmental deformities at 48 h post-treatment. The ** and *** express the statistical difference between the groups at *p* < 0.01 and *p* < 0.001, compared to CML + PBS for percentage hatching, while # and ### express the statistical difference at *p* < 0.05 and *p* < 0.001, compared to CML + PBS for percentage developmental deformities. (**E**) Quantification of the DHE and (**F**) AO fluorescent intensity using Image J-based software version 1.53r. *, **, and *** express the statistical difference at *p* < 0.05, *p* < 0.01, and *p* < 0.001, respectively, compared to CML + PBS group; ns denoting the non-significant difference between the group.

As depicted in Figure 6, introducing CML (500 ng) through microinjection into zebrafish embryos led to the lowest survival rate (24 ± 2% survivability) at 24 h post-injection (Figure 6A). Conversely, injecting PBS alone exhibited the highest survivability, approximately 68 ± 3%. In the presence of CML, a co-injection of rHDL-(1:1) yielded significantly higher embryo survivability (~52 ± 3%) (*p* < 0.001 versus CML + PBS). In contrast, among rHDL groups, co-injecting with rHDL-(1:0) resulted in lower survivability (~35 ± 3%, *p* = 0.041 versus CML + PBS). Spearman correlation analysis showed that the survivability was positively correlated with an increase in CIGB-258 content in the rHDL (*r* = 0.980, *p* = 0.020) because the rHDL-(1:0.1) and rHDL-(1:0.5) injected groups showed 40 ± 2% and 44 ± 3%, respectively.

The stereo image of the embryos showed that the PBS-alone group had a normal developmental morphology at 48 h post-treatment (Figure 6B) with the highest number of somites (35 ± 1) and embryo hatching (75.5%) (Figure 6C,D). Also, the least developmental deformities (21.5%) were observed in the embryos of the PBS-alone group (Figure 6B,D). Conversely, the embryo injected with CML-alone displayed pronounced embryonic developmental defects (94%), with no sign of somite development, and the least embryo hatching (5%) at 48 h post-treatment (Figure 6B–D). The co-injection of different rHDLs displayed improved embryo survivability and restoration of CML-impaired morphological changes (Figure 6B–D). In the rHDL-(1:0) injected group, a 24 ± 1 somite and 23% embryo hatching were observed, which is substantially better than the embryo hatching observed in only the CML injected group. Nonetheless, most of the embryos (84%) exhibited developmental deformities. In contrast to rHDL-(1:0), the embryos injected with rHDL containing CIGB-258 [i.e., rHDL-(1:0.1), rHDL-(1:0.5) and rHDL-(1:1)] displayed a dose-dependent effect on the improvement of embryo hatching and the restoration of developmental deformities provokes by the CML. The embryos that received the rHDL-(1:0.5) treatment showed 52% embryo hatching and 41% developmental deformities, which is significantly 10.4-fold higher and 2.2-fold lower, respectively, than the embryo hatching and developmental deformities observed in CML + PBS injected group. The most noteworthy results were observed in the rHDL-(1:1) injected group, evident by higher (60%) embryo hatching with a marked increase in the somite numbers (32 ± 1) at 48 h post-treatment. Also, only 38% of developmental deformities in the embryos at 48 h post-treatment were observed in the rHDL-(1:1)-injected group. While compared to the CML + PBS-injected group, the rHDL-(1:1)-injected group displayed a 12-fold higher embryo hatching and 2.5-fold lower embryo developmental defects at 48 h post-treatment, signifying the importance of CIGB-258 to prevent the adverse effect exerted by the CML.

Acridine orange (AO) and dihydroethidium (DHE) staining showed that the CML + PBS group exhibited the most pronounced green and red intensities, respectively. This implies that the introduction of CML resulted in the highest degree of apoptosis and ROS production (Figure 6B,E). Conversely, the CML + rHDL-(1:1) group displayed the least intense red and green signals, indicating that the concurrent presence of CIGB-258 in rHDL suppressed ROS level and mitigated cellular apoptosis.

In particular, (as shown in Figure 6B,E), AO staining to perceive cellular apoptosis revealed that the CML alone group experienced a 3.2-fold greater increase in apoptosis compared to PBS group. This observation suggests that the injection of CML induced acute cell death. In contrast, co-injection of rHDL-(1:1) resulted in the lowest level of apoptosis (~54% less than the CML alone group). The rHDL-(1:0) group did not exhibit a significant reduction, showing only an ~11% decrease compared to the CML + PBS group. Notably, the rHDL-(1:0.1) and rHDL-(1:0.5) groups demonstrated a more substantial decrease in apoptosis (around 27% and 42%, respectively) compared to the CML + PBS group.

DHE staining was employed for ROS detection, revealing a 3.4-fold surge in ROS production induced by CML compared to the PBS alone group (Figure 6B,F). Co-injection of rHDL-(1:0) exhibited the least efficacy in reducing ROS production by approximately 23% decrease compared to the CML alone group. Conversely, co-injection of rHDL-(1:1) demonstrated the most robust activity, leading to a substantial ~63% reduction in ROS generation. The co-injection of rHDL-(1:0.1) and rHDL-(1:0.5) exhibited comparable ROS production, resulting in ~45% lower ROS level than the CML + PBS group. In summary, all rHDL variants displayed sufficient protective efficacy against CML toxicity in a concentration-dependent manner of CIGB-258. Notably, rHDL-(1:1) exhibited the highest potency in promoting survivability and inhibiting developmental deformities. 

### 2.7. Anti-Inflammatory Activity against Neurotoxicity of CML

**Figure 7:**
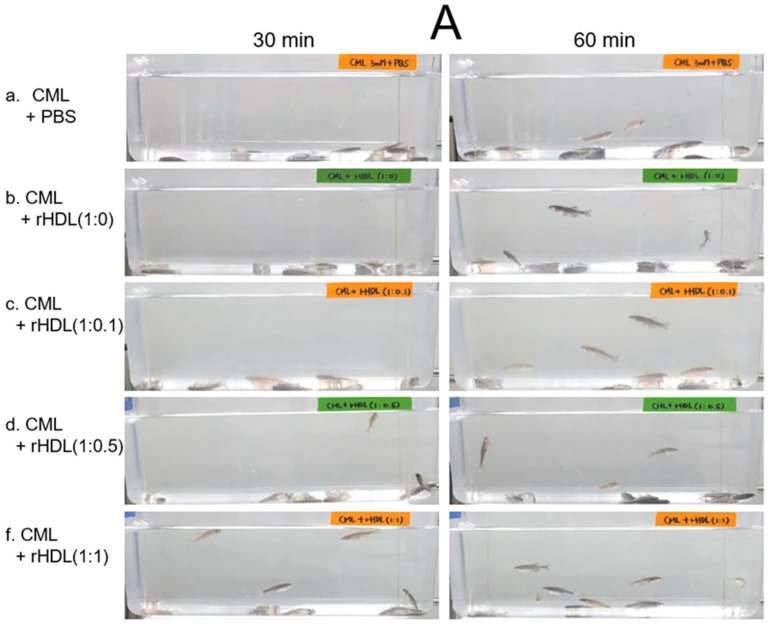

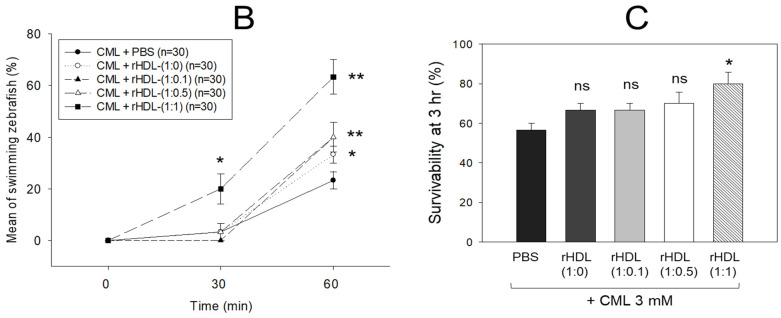
Investigation into the impact of reconstituted high-density lipoproteins (rHDL) with CIGB-258 on carboxymethyllysine (CML)-induced impairment of swimming behavior and mortality of adult zebrafish. (**A**) Evaluation of various rHDL formulations against CML-induced impairment of swimming activity in adult zebrafish at 30 and 60 min post-treatment. (**B**) Percentage of swimming zebrafish at 30 min and 60 min post-injection of CML and rHDLs. * and ** express the statistical difference at *p* < 0.05 and *p* < 0.01, respectively, compared to only the CML injected group. (**C**) Assessment of varied rHDL impact on CML-induced mortality in adult zebrafish over a 3 h post-treatment. * signify the statistical difference at *p* < 0.05, compared to only the CML injected group, ns denoting the non-significant difference between the groups.

An intraperitoneal (IP) injection of CML induced acute paralysis in adult zebrafish within 30 min post-injection. In the CML-alone group (depicted in photograph a), all zebrafish exhibited an inability to swim, lying on the tank bottom with occasional quivering (Figure 7A). Despite being still alive, they displayed tremors at 30 min post-injection. By 60 min post-injection, 30% of the fishes in the CML-alone group regained swimming capabilities, with a survival rate of 60 ± 5%. However, their swimming pattern was characterized by wobbling, seizures, and uncontrollable movements (Supplemental Video S1). The CML + rHDL-(1:0) group exhibited a gradual recovery in swimming ability; approximately 33% of fish resumed swimming at 60 min post-injection (Figure 7B) and a survivability of 67 ± 8% (Supplemental Video S2). On the other hand, the group treated with CML + rHDL-(1:1) exhibited the most substantial improvement in swimming capability, manifesting a more vigorous and instinctive swimming pattern (Supplemental Video S5). Approximately 63 ± 7% of the fish could swim again 60 min post-injection, with 86 ± 6% survivability. Similarly, the rHDL-(1:0.1) and rHDL-(1:0.5) groups showed an improved recovery of their swimming ability (~40 ± 5%) at 60 min post-injection with 76 ± 8% survivability (Supplemental Videos S3 and S4).

As shown in Figure 7C, at 3 h post-injection, the CML + PBS group showed the lowest survivability of approximately 56 ± 3%, while a co-injection of rHDL-(1:0) increased the survivability to approximately 67 ± 3%. Interestingly, the rHDL-(1:0.1) and rHDL-(1:0.5) group showed 67 ± 3% and 70 ± 5% survivability, respectively, showing similar or slightly higher anti-inflammatory activity than rHDL-(1:0). On the other hand, the rHDL-(1:1) group showed the highest survivability (~80 ± 5%), suggesting that the incorporation of CIGB-258 improved the anti-inflammatory activity to neutralize the CML toxicity in a dose-dependent manner. 

### 2.8. Impact of rHDL on the Blood Lipid Profile

**Figure 8:**
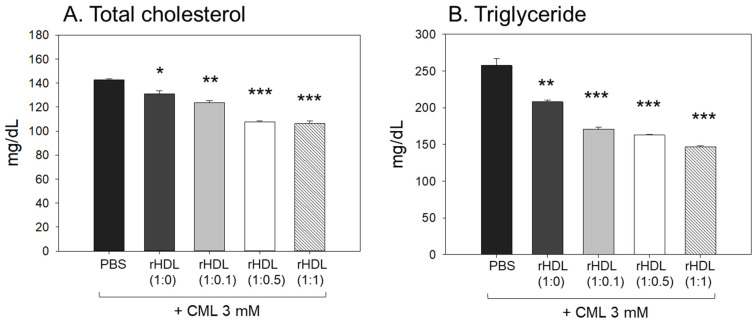
Assessment of serum (**A**) total cholesterol (TC) and (**B**) triglyceride (TG) in zebrafish plasma following exposure to carboxymethyllysine (CML) in the presence of different formulations of reconstituted high-density lipoproteins (HDL). Quantification of TC and TG levels were performed 180 min-post injections. *, **, and *** signify the statistical difference at *p* < 0.05, *p* < 0.01, and *p* < 0.001, respectively, compared to the CML + PBS-injected group.

Following the extraction of plasma from each zebrafish group, the analysis of plasma lipids revealed that the CML+ PBS group exhibited the highest levels of total cholesterol (TC) and triglyceride (TG) plasma levels, as illustrated in Figure 8A,B. In particular, the serum TG level in the CML group increased approximately 2.0-fold compared to the PBS-alone group, which was higher than that of TC. An injection of CML caused an abrupt inflammatory response with an abrupt increase in serum TG. While all rHDL had adequate lipid-lowering effects in a dose-dependent manner, the CML + rHDL-(1:1) group exhibited the minimal TC and TG among the groups, 26% and 44% lower, respectively, than that of the CML + PBS group. The serum TG levels were reduced further by a co-injection of rHDL than that of the TC levels, suggesting that the elevation in inflammation was more associated with the increase in TG levels. 

### 2.9. Serum AST and ALT after Exposure with rHDL

**Figure 9:**
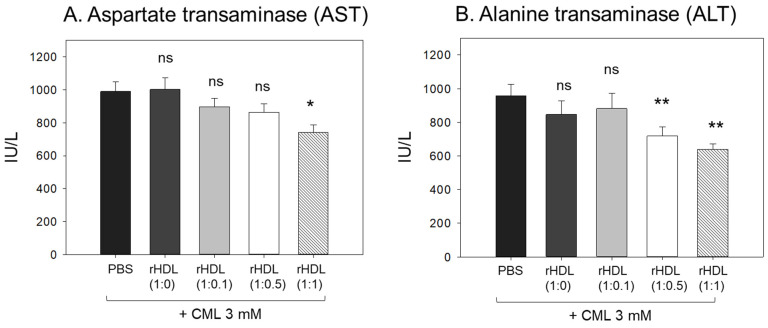
Quantification of hepatic enzymes (**A**) aspartate transaminase (AST) and (**B**) alanine transaminase (ALT), in zebrafish plasma. All zebrafish were sacrificed at 180 min post injections of CML and each rHDL. *, *p* < 0.05 vs. CML + PBS; **, *p* < 0.01 vs. CML + PBS. AST, aspartate aminotransferase; ALT, alanine aminotransferase; CML, carboxymethyllysine; rHDL, reconstituted high-density lipoproteins.

The CML + PBS group showed the highest AST and ALT levels (approximately 989 IU/L and 958 IU/L, respectively) (Figure 9), while the CML + rHDL-(1:1) group showed the lowest AST and ALT levels (approximately 741 IU/L and 638 IU/L, respectively). The rHDL-(1:0) and rHDL-(1:0.1) groups showed similar AST and ALT levels to the CML + PBS group (897–1002 IU/L and 846–880 IU/L, respectively). Interestingly, the rHDL-(1:0.5) group showed similar AST levels to the CML + PBS group but 25% lower ALT levels than the CML + PBS group. Hence, a co-injection of rHDL containing CIGB-258 ameliorated the hepatic damage, especially lowering the ALT levels caused by the CML injection in a dose-dependent manner.

### 2.10. Histologic Analysis of Hepatic Tissue

**Figure 10:**
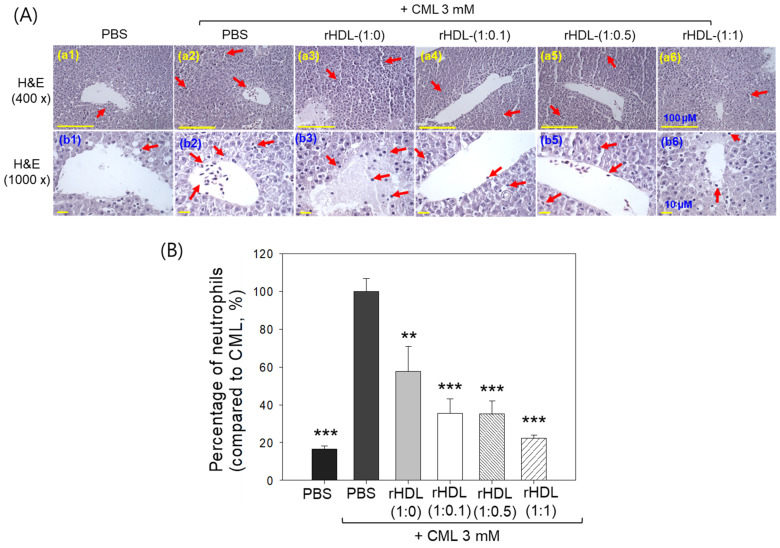
Histological examination of the hepatic tissue from zebrafish subjected to CML and each rHDL treatment. (**A**) Illustrations (**a1**–**a6**) display the hematoxylin and eosin (H&E) stained region of the hepatic section at 400× magnification [scale bar = 100 μm]. Images (**b1**–**b6**) depict the 1000× magnified views in the vicinity of the portal vein within the H&E-stained area [scale bar = 10 μm]. Red arrow indicates infiltrated neutrophils. (**B**) The graphs represent the percentage of the neutrophils observed in the H&E-stained area. The ** and *** express the statistical difference at *p* < 0.01 and *p* < 0.001, respectively, compared to CML + PBS; ns denoting the non-significant difference between the groups.

Liver histology analysis through H&E staining revealed that the group treated with CML + PBS (Figure 10A, photographs a2 and b2) exhibited the highest neutrophil count, considered as 100% (Figure 10B). Conversely, the CML + rHDL-(1:1) group (photograph a3 and b3) demonstrated a significantly reduced neutrophil count (57.7 ± 13.0%), which was 1.7-fold lower than that quantified in the CML + PBS group. This suggests that rHDL-(1:1) may effectively exert an anti-inflammatory effect against CML-induced inflammation. In contrast, the CML + rHDL-(1:0) group showed higher neutrophil counts, while the CML + rHDL-(1:0.1) (photographs a4 and b4) and CML + rHDL-(1:0.5) (photograph a5 and b5) groups exhibited substantially reduced neutrophil numbers (Figure 10A,B). Notably, the most promising results were observed in rHDL-(1:1) around 22.2 ± 1.8%, which was significantly 4.5-fold lower than the neutrophil counts in the CML + PBS-injected group. These findings indicate that incorporating CIGB-258 enhances the anti-inflammatory activity of rHDL in a dose-dependent manner.

### 2.11. Immunohistochemical Assessment of Hepatic Tissue for IL-6 Analysis

**Figure 11:**
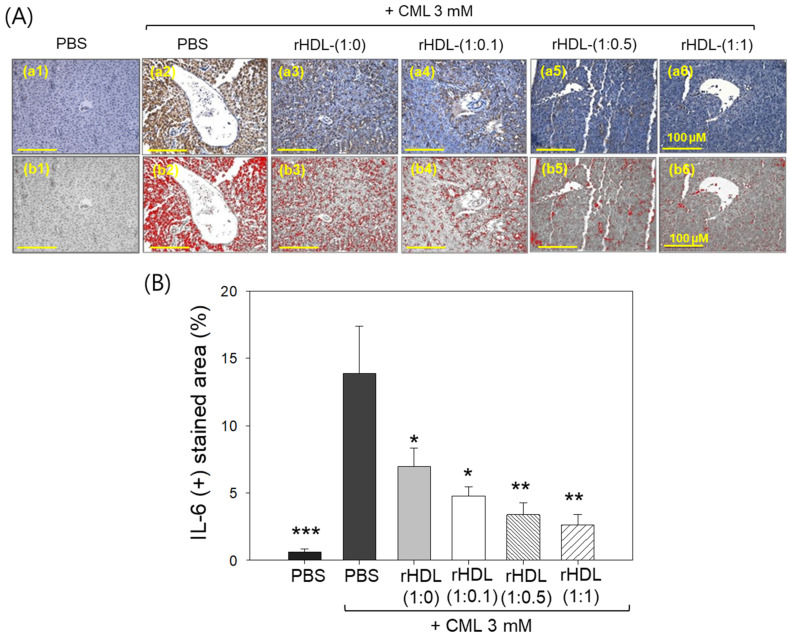
Immunohistochemistry (IHC) observations for the detection of interleukin (IL)-6 level in the liver samples of zebrafish injected with carboxymethyllysine (CML) and reconstituted high-density lipoprotein (rHDL). (**A**) Images (**a1**–**a6**) depict typical IHC representations utilizing IL-6 antibody-stained liver tissue. Images (**b1**–**b6**) correspond to the IHC stained area where brown color has been substituted with red color [at a threshold value of (20–100)] using Image J software to enhance the visualization of the stained area. The red color-stained area (corresponding to the IL-6 IHC stained area) was calculated using Image J software. The yellow scale bar denotes 100 μm. (**B**) Graph shows the percentage quantification of the IL-6-stained area. The *, **, and *** express the statistical difference at *p* < 0.05, *p* < 0.01, and *p* < 0.001, respectively, compared to only the CML-injected group.

Immunohistochemical assessment of interleukin (IL)-6 in liver tissue demonstrated that the CML alone group exhibited the highest stained area constituting ~13.8%, while the CML + rHDL-(1:1) group displayed the minimum IL-6-stained area of only 2.6% (Figure 11A). Notably, the rHDL-(1:0), rHDL-(1:0.1), and rHDL-(1:0.5) groups exhibited a dose-dependent reduction in the IL-6-stained area, evident by 6.9%, 4.7%, and 3.4% stained area, respectively, i.e., 2.0-, 2.9-, and 4.1-fold lower, than the IL-6-stained area appeared in the CML + PBS group. In Figure 11B, it is evident that the CML + rHDL-(1:1) group demonstrated the least-IL-6-stained area (2.6%), which is 5.3-fold (*p* < 0.01) lower than the IL-6-stained area noticed in CML + PBS group, underscoring the potent anti-inflammatory activity associated with concurrent presence of CIGB-258. 

### 2.12. Assessment of ROS Generation and Apoptosis in Liver Tissue

**Figure 12:**
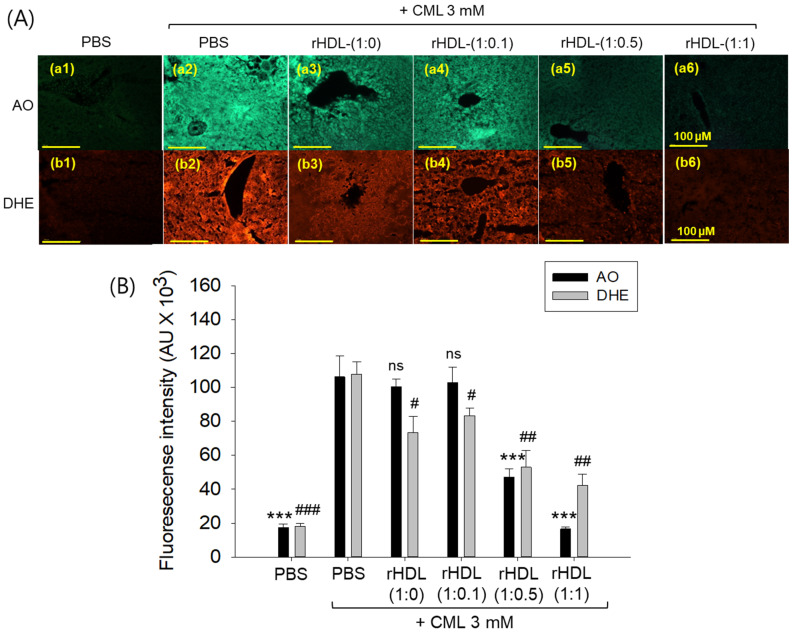
Effect of various rHDL against the carboxymethyllysine (CML)-induced reactive oxygen species (ROS) and extent of apoptosis in the hepatic tissue. (**A**) Images (**a1**–**a6**) represent the acridine orange (AO) staining to evaluate the extent of apoptosis, while images (**b1**–**b6**) represent the dihydroethidium (DHE) staining to evaluate the ROS production [scale bar, 100 μm]. (**B**) Quantification of AO and DHE stained area employing Image J software. The *** expresses the statistical difference at *p* < 0.001, compared to the CML + PBS group for AO-stained area. #, ##, and ### express the statistical significance at *p* < 0.05, *p* < 0.01 and *p* < 0.001, respectively, compared to CML + PBS for DHE stained area. The ns denoting the non-significant difference between the groups.

AO staining revealed that the CML + PBS group exhibited the highest green fluorescence, indicating heightened levels of cellular apoptosis (Figure 12A). This suggests that intraperitoneal injection of CML induces acute apoptosis. Conversely, CML + rHDL-(1:1) group demonstrated the lowest level of apoptosis, approximately 85% lower (*p* < 0.001) than the CML + PBS group. While the rHDL-(1:0) and rHDL-(1:0.1) groups displayed a similar extent of apoptosis to the CML + PBS group, the rHDL-(1:0.5) group exhibited a 56% reduction of apoptosis (Figure 12B). These results suggest that co-injection of rHDL containing CIGB-258 could protect hepatic cells from apoptosis caused by CML-toxicity in a dose-dependent manner of CIGB-258. 

DHE staining revealed that the CML alone group exhibited the most elevated red fluorescence intensity, indicating the highest production of ROS. Conversely, co-treatment with rHDL resulted in a reduction of red intensity. Within the various rHDL groups, the CML + rHDL (1:1) combination displayed the least ROS production, approximately 60% lower (*p* < 0.01) than the CML + PBS group. Although the rHDL-(1:0) group and rHDL-(1:0.1) group showed similar inhibition of ROS production (23–32% lower ROS production (*p* < 0.05) than the CML + PBS group), the rHDL-(1:0.5) group a showed 52% reduction in ROS production (Figure 12B). Hence, all rHDL containing CIGB-258 exerted considerable inhibition against ROS production in a dose-dependent manner in the presence of CML. Overall, incorporating CIGB-258 in rHDL showed the remarkable protection of hepatic tissue from cellular apoptosis and ROS production in a dose-dependent manner of CIGB-258. 

## 3. Discussion

The enhancement of apoA-I is an important strategy to improve the HDL particle quality and functionality and maximize its therapeutic activity [18]. Many site-directed mutagenesis of apoA-I and natural variants of apoA-I showed the importance of the apoA-I structure to enhance the HDL functionality [19]. Among them, point mutants of apoA-I, such as R173C and V156K, revealed enhanced structure and functionality to exert anti-inflammatory activity and regression effects and remove preexisting atherosclerotic plaques [20]. On the other hand, the over-production of apoA-I variants and the synthesis of rHDL to enforce HDL functionality have a significant hurdle practically because of the time-consuming and expensive cost via high-dose intravenous blood infusion of around 2–6 g of HDL [21]. Therefore, a more efficient and economical strategy is needed to reinforce HDL stability and functionality, such as encapsulating hydrophobic drugs and peptides.

CIGB-258, known an altered peptide ligand (APL), comprises 27 amino acids with a molecular weight of 2987. It is derived from HSP60, a 60 kDa chaperone identified as heat shock protein family D (HSPD) member 1 [22]. HSP60 is involved in various molecular functions within the toll-like receptor signaling pathway via apoA-I/HDL binding, ATP binding, protein folding and stabilization, and T-cell activation [23,24]. In the present investigation, apoA-I and CIGB-258 demonstrated strong binding, resulting in the formation of rHDL and causing a blue shift in the WMF of apoA-I. This suggests the stabilization of the tertiary structure of apoA-I as illustrated in Figure 1. As the CIGB-258 content was increased, the synthesized discoidal rHDLs showed an increase in particle size (Figure 2) and resistance to isothermal denaturation (Figure 3). The rHDL-(1:1) group exhibited the strongest antioxidant ability against cupric ion-mediated LDL oxidation (Figure 4) and anti-glycation activity to protect HDL from CML-mediated proteolytic degradation of apoA-I (Figure 5). CML has been recognized for its harmful effects due to the generation of ROS and oxidative stress [25], triggering the apoptosis, Additionally, there is evidence supporting the active involvement of CML as a regulator of apoptosis, notably affecting caspase-3 and caspase-9 [26]. The rHDL containing CIGB-258 helped to prevent CML-mediated embryo death with the suppression of ROS generation and cellular apoptosis in zebrafish embryo (Figure 6). 

In the current study, rHDL revealed potent anti-inflammatory activity against CML-mediated proinflammatory neurotoxicity and paralysis as the CIGB-258 content was increased (Figure 7). The rHDL-(1:1) group showed the lowest serum TC, TG, AST, and ALT levels (Figure 8 and Figure 9), as well as the least neutrophil infiltration (Figure 10), IL-6 production (Figure 11), ROS production, and cellular apoptosis in the liver (Figure 12). Regarding the therapeutic importance of this study, therapies that mimic HDL function, including rHDL with apoA-I and CIGB-258, addressed to increase their stability and functionality, constitute promising treatment strategies on the three important and widespread pathologies: atherosclerosis, Alzheimer’s disease, and diabetes mellitus. 

Interestingly, the incorporation of CIGB-258 could increase the particle size of rHDL with a more stabilizing tertiary structure of apoA-I against urea-induced denaturation, suggesting that adding the peptide could enhance the apoA-I stability. Many studies showed that the incorporation of apolipoproteins, such as apoA-II, apoC-III, serum amyloid A (SAA)-1, and α-synuclein (α-syn), in rHDL, resulted in a negative change in the structural stability and functionality [14,16,27]. The addition of apolipoprotein A-II (apoA-II) in rHDL containing apoA-I resulted in an almost 50% displacement of apoA-I, around an apoA-I:apoA-II molar ratio of 1:1, resulting in the destabilization of apoA-I and modulation of lecithin:cholesterol acyltransferase (LCAT) activity [28]. The enrichment of human apoA-II also displaced paraoxonase (PON) from HDL and impaired the antioxidant activity in apoA-II transgenic mice [29]. In addition, an increase in apoC-III in rHDL impaired several beneficial properties of HDL, including decreased particle size and apoA-I numbers in rHDL with a reduced antioxidant ability against LDL oxidation [30]. Furthermore, the co-presence of serum amyloid A (SAA) and apoA-I in rHDL resulted in a decrease in particle size and an impairment of the structural stability with a 3 nm red-shift of the WMF [14], indicating that Trp108 was more exposed to the polar phase. These reports suggest that the amphipathic a-helix domain of similar apolipoproteins could disturb the normal movement of the middle mobile helix domain. The amphipathic helices within apoA-I are thought to play a role in the structural rearrangements of HDL as their lipid and apolipoprotein compositions undergo changes during metabolism. A region referred to as “hinge” or “mobile region” spanning residues 100 and 183 of apoA-I, specifically the central mobile helix domain, is implicated in these rearrangements. The change in WMF originates from Trp in apoA-I because CIGB-258 has no Trp. Interestingly, an increase in SAA content in the rHDL containing apoA-I accelerated apoA-I glycation [14], but the current and previous results showed that an increase of CIGB-258 content in rHDL containing apoA-I resulted in the remarkable inhibition of apoA-I and HDL glycation [5,6]. As shown in Figure 5, proteins and amino acids glycated through CML treatment exhibited dynamic characteristics, as microorganisms are capable of breaking down these compounds [31,32]. It has been postulated that CML could potentially engage with Lys and Arg residues of apoA-I, destabilizing both apoA-I and HDL structures. The compromised structure of apoA-I has been associated with the diminished functionality of HDL, including a reduction in antioxidant capability, anti-microbial activity, and susceptibility to proteolytic degradation.

Although several peptides and proteins induce severe impairment of the structure and functions of apoA-I, such as SAA-1 [14], β-amyloid [16], and α-synuclein [27], no study has shown that a peptide/protein can enforce apoA-I/HDL functionality via structural stabilization. As far as the authors know, this study represents the initial report that CIGB-258 could stabilize and enhance structural and functional correlations of apoA-I and HDL. Interestingly, CIGB-258 and SAA are enriched with α-helix content, 81% and 66%, respectively, in the entire sequence, while apoA-I is also enriched with an α-helix, approximately 76%. Furthermore, CIGB-258 and SAA have a similar isoelectric point (pI) (5.912 and 6.341, respectively), which is also similar to apoA-I (6.0–6.4). These results suggest that CIGB-258 and SAA-1 display similar secondary and tertiary structures. On the other hand, upon binding with apoA-I, their behavior was remarkably different in rHDL. The incorporation of SAA into rHDL had a detrimental effect on the HDL structure and functions by increasing glycation in apoA-I and HDL [14], indicating an impairment of the apoA-I helix domain structure by SAA. Nevertheless, incorporating CIGB-258 into rHDL helped protect the HDL structure and functions by inhibiting glycation in apoA-I and HDL. Although the precise mechanism is still unclear, these differences between CIGB-258 and SAA might contribute to the dramatic difference in the anti-inflammatory (CIGB-258) and proinflammatory (SAA-1) properties in the physiological state. 

The acute phase apolipoprotein, human SAA1, has been detected in the amyloid deposits within the tissue of individuals experiencing chronic or recurrent inflammation [33,34]. An increased serum SAA level serves as a biomarker for atherosclerosis and sepsis [35,36] due to its role in destabilizing HDL and displacing apoA-I, leading to the dysfunctionality of HDL. By contrast, the current results showed that CIGB-258, like exchangeable apolipoproteins, could associate well with apoA-I to form larger rHDL particles and enhance the antioxidant, anti-glycation, and anti-inflammatory functions of apoA-I/HDL. These findings extend the therapeutic use of CIGB-258 for chronic inflammatory diseases characterized by increased serum SAA such as sepsis, diabetes, and cardiovascular diseases. 

Interestingly, Dominguez Horta et al. showed that CIGB-258 could bind to apoA-I using CIGB-258 coupled affinity chromatography [37], suggesting that CIGB-258 has a specific affinity to apoA-I via a putative interaction of the amphipathic helix domains. In the present study, it has been shown that the binding of CIGB-258 to apoA-I exerts protective action in the rHDL context and contributes to the structural and functional enhancement of apoA-I and HDL. These results reinforce the therapeutic potential of CIGB-258 for the treatment of diseases associated with disorders in lipid homeostasis. These findings are congruent with the in vivo anti-inflammatory activities of CIGB-258 in autoimmune diseases [38] and acute infectious diseases, specifically rheumatoid arthritis (RA) [22]. In fact, this peptide exerted a potent therapeutic effect in experimental models of RA [39,40], and this effect was corroborated in clinical investigations in patients with RA by subcutaneous administration [41,42,43]. In addition, the effect of CIGB-258 in acute infectious diseases, especially in suppressing the cytokine cascade and controlling neutrophil activation, was demonstrated in hyperinflammatory COVID-19 patients [44].

On the other hand, during sepsis, SAA could displace apoA-I from the HDL surface, generating free apoA-I, which is removed faster by the kidney. Therefore, the quantity and quality of HDL were severely damaged by the removal of apoA-I, resulting in the loss of anti-inflammatory properties [45,46]. These results suggest that CIGB-258 could be a candidate to treat acute inflammatory diseases, such as sepsis, by stabilizing apoA-I and inhibiting the displacement of apoA-I by SAA. Future study is necessary to investigate a putative effect of CIGB-258 to protect apoA-I structure in the lipid-free state and HDL state under acute phase stress, such as oxidation, glycation, and displacement by SAA.

This study had some limitations, such as the in vitro synthesis of rHDL with only apoA-I and CIGB-258 at designated three molar ratios, 1:0.1, 1:0.5, and 1:1, which were not optimized. Therefore, the physiological distribution and binding affinity of CIGB-258 with apoA-I in the blood HDL should be identified to find the optimum molar ratio of apoA-I:CIGB-258. Future studies are needed to elucidate the following: (1) which helix domain of apoA-I is involved in interacting with CIGB-258; (2) the relative specific binding affinity of apoA-I to CIGB-258 and apoA-I to SAA; (3) the effect of the co-presence of CIGB-258, SAA, and apoA-I in rHDL regarding the structural and functional correlations; and 4) the putative protective effect of CIGB-258 against displacing apoA-I by SAA in HDL. These results reinforce the protective capacity of CIGB-258 on apoA-I and constitute a promising strategy for acute and chronic inflammatory diseases therapy. However, further study in higher vertebrate animals and larger clinical trials are needed to better assess the future role of rHDL containing CIGB-258 in the global management of diseases associated with disorders in lipid homeostasis.

## 4. Materials and Methods

### 4.1. Materials

Jusvinza^®^ (CIGB-258) is a lyophilized powder formulation containing synthesized peptide derived from HSP60, comprising 27 amino acids (1.25 mg/vial, Lot# 1125J1/0). The peptide was acquired from the Center for Genetic Engineering and Biotechnology (CIGB) in Havana, Cuba with the compliance to be utilized exclusively for research purposes. All other chemicals and reagents, unless specified otherwise, were of analytical grade and used as supplied.

### 4.2. Lipoproteins Isolation

The lipoproteins (LDL and HDL) were extracted from human blood through density gradient ultracentrifugation [47]. Blood donation was obtained from voluntary human participants (25 ± 3 years) who underwent 16 h fast, following the Helsinki guidelines approved by the Institutional Review Board of Korea National Institute for Bioethics Policy (KoNIBP; authorization number P01-202109-31-009). The 10 mL of blood was centrifuged (4000× *g*, for 20 min) to obtain the serum. Subsequently, 3 mL of the serum was suspended on the density gradient mixture prepared by NaCl (1.019 < d < 1.063) and NaBr (1.063 < d < 1.225) and subjected to overnight ultracentrifuge (Himac NX, Hitachi, Tokyo, Japan) at 100,000× *g*. The separated LDL (1.019 < d < 1.063) and HDL (1.063 < d < 1.225) were recovered from their respective density zones and subjected to individual dialysis using Tris-buffered saline (pH 8.0). The dialyzed LDL and HDL were recovered and kept in the refrigerator for further use.

### 4.3. ApoA-I Purification

The apoA-I was extracted from the HDL following the earlier described method [48]. In brief, 5 mg of HDL was mixed with 1 mL of chloroform and methanol solution (1:1 *v*/*v*) and vortexed. The delipidated apoA-I underwent purification through a Superose 6 10/300 GL column (GE Healthcare) employing fast protein liquid chromatography, facilitated by an AKTA purifier system (GE Healthcare, Uppsala, Sweden) using 10 mM Tris-HCl/140 mM NaCl (pH 8.0) solvent system. The purity of the separated ApoA-I was assessed through SDS-PAGE.

### 4.4. Formulation of Reconstituted HDL and Electrophoresis

The reconstituted HDL (rHDL) was constructed using phospholipids, cholesterol, apoA-I and CIGB-258 following the earlier-described method [49]. Palmitoyloleoyl phosphatidylcholine (POPC) served as the phospholipid source. The synthesis of rHDL commenced by combining POPC and cholesterol in chloroform-methanol (1:1 *v/v*) solution in the specified ratios, as mentioned in Table 2. The mixture was mixed and evaporated under a gentle stream of nitrogen. Subsequently, Tris-buffered saline (TBS, pH 8.0) and a sodium cholate solution (30 mg/mL) were added along with apoA-I (1 mg/mL) and CIGB-258 (2.5 mg/mL), to achieve the final volume 0.7 mL with a ratio specified in Table 2. The resulting content underwent dialysis with TBS (pH 8.0) for 18 h at 4 °C to eliminate the sodium cholate. The synthesized rHDL was then subjected to electrophoresis on SDS-PAGE (15%) and 0.5% agarose gel. Band intensity (BI) was determined by using Chemi-Doc^®^ XR (Bio-Rad, Hercules, CA, USA) and Quantity One software (version 4.5.2).

### 4.5. Trp Fluorescence Characterization during Isothermal Denaturation

The wavelengths of maximum fluorescence (WMF) of tryptophan (Trp) in apoA-I were determined using a previously described method [14,16]. In brief, samples were excited at 295 nm to prevent interference from tyrosine fluorescence, and the emission spectra were recorded from 305 to 400 nm. Isothermal denaturation experiments were conducted by assessing the impact of urea concentration (0 M to 7 M) on the secondary structures of apoA-I and CIGB-258 in both lipid-free and lipid-bound states. The Trp movement was measured using fluorospectroscopy, following the earlier reported procedures [14].

### 4.6. Transmission Electron Microscopic (TEM) Analysis

The size of the synthesized rHDL particles was assessed by TEM analysis as outlined in a previously described method [4]. In brief, 10 μL of rHDL sample was mixed with 10 μL of 1% sodium phosphotungstate (PTA, pH 7.4). Following this, 6 μL of the resulting suspension was applied to a Formvar carbon-coated 300 mesh copper grid and examined using a TEM (Hitachi, model HT-7800; Tokyo, Japan) at 80 kV (accelerating voltage) and 150,000× magnification. 

### 4.7. Oxidation of LDL and Inhibition by CIGB-258 in rHDL

The ability of each rHDL to prevent LDL oxidation was assessed by agarose gel electrophoresis [50] and thiobarbituric acid reactive substances (TBARS) assay [51]. In brief, LDL (2 mg/mL) was incubated with CuSO4 (10 μM) for 4 h at 37 °C, both in the presence and absence of HDL. Subsequently, the samples underwent electrophoresis (0.5% agarose gel at 50 V) to determine the extent of oxidation. The gel was stained with 1.25% Coomassie brilliant blue to analyze the extent of the LDL’s oxidation and apolipoprotein-B (apo-B). Additionally, the lipid peroxidation of LDL was assessed using the TBARS assay [51] by quantifying the malondialdehyde (MDA) level.

### 4.8. Anti-Glycation Activity of CIGB-258 in rHDL

The assessment of the antiglycation effect involved incubating different rHDL at 1 mg/mL (50 μL) with 2 mg/mL of native HDL (300 μL) in the presence of 400 μM CML (150 μL). Subsequently, 100 μL of a 0.2 M KH_2_PO_4_/0.02% NaN_3_ buffer (pH 7.4) was added, and the content was incubated at 37 °C for 96 h. The anti-glycation effect was evaluated through SDS-PAGE (15%) (5 μg HDL from each sample was loaded) following Coomassie brilliant blue staining and densitometric analysis. The degree of glycation was assessed by a significant reduction in the protein content of HDL (apoA-I), determined by the quantifying band intensity (BI) using Chemi-Doc^®^ XR (Bio-Rad) and Quantity One software (version 4.5.2). The fluorescence of HDL (containing apoA-I) + CML under various rHDL treatments was measured at an excitation wavelength of (370 nm) and an emission wavelength of (440 nm) to assess the degree of advanced glycation reactions [6,52].

### 4.9. Protein Determination in Lipoprotein

The protein quantification in HDL, LDL and rHDL was determined by the Lowry method with a slight modification reported by Markwell et al. [53]. Bovine serum albumin (BSA) served as a standard. Lipid-free apoA-I protein quantification was achieved by Quick Start^TM^ Bradford Protein Assay Kit (Bio-Rad #5000201) employing Bovine serum albumin (BSA) as a reference.

### 4.10. Zebrafish Maintenance, Mating and Embryo Production

Standard protocol [54] was followed for the maintenance of zebrafish and their embryos in accordance with the Guide for the Care and Use of Laboratory Animals [55]. The procedure was approved by the Committee of Animal Care and Use of Raydel Research Institute (approval code RRI-20-003, Daegu, Republic of Korea). Zebrafish embryo production involved segregating male and female zebrafish in a 2:1 ratio within the breeding tank using a physical barrier. After 16 h of isolation, the barriers were removed, allowing uninterrupted mating for approximately 1 h. Subsequently, embryos were collected and washed with water.

### 4.11. Infusion of CML and the rHDL into Zebrafish Embryos

Zebrafish embryos at 1 h post-fertilization (hpf) underwent individual microinjections utilizing a pneumatic picopump (PV830; World Precision Instruments, Sarasota, FL, USA). The injection consisting of rHDL alone (400 pg of apoA-I) or co-injection with CML (500 ng) occurred in the same location to reduce bias, following an earlier described method [5,6]. Immediately post-injection and 5 h later, the microscopic examination of the embryos was evaluated, and images were captured at 10× and 20× magnification using a ZEISS Axiocam 208 colour (Jena, Germany). Subsequently, at 24 h and 48 h post-injection, morphological changes in the embryos were visualized under a microscope.

### 4.12. Oxidative Stress and Apoptosis Evaluation in the Embryo

The investigation of reactive oxygen species (ROS) and apoptosis in embryos injected with CML and co-treated with rHDL were examined using dihydroethidium (DHE) and acridine orange (AO) fluorescent staining, a previously outlined method [56,57]. In brief, embryos were immersed in 500 μL of DHE (30 μM) and incubated for 30 min in the dark. After two washes with distilled water, embryos were observed under a fluorescent microscope with an excitation wavelength of 585 nm and an emission wavelength of 615 nm. 

Likewise, embryos were immersed in 500 μL of AO (5 μg/mL) for 30 min, followed by two washes with PBS. Subsequent visualization occurred under fluorescence microscopy at emission and excitation wavelengths of 505 nm and 535 nm, respectively. The fluorescent images of DHE and AO-stained embryos underwent processing for the fluorescent intensity quantification using Image J software version 1.53r (http://rsb.info.nih.gov/ij/, accessed on 11 July 2023).

### 4.13. Anti-Inflammatory Effect of rHDL in Zebrafish

An acute inflammation in zebrafish was induced by the intraperitoneal injection of carboxymethyllysine (CML 250 μg in 10 μL of PBS), i.e., corresponding to 3 mM CML (considering 300 mg as the average body weight of zebrafish) [5]. The zebrafish were randomly allocated into five cohorts (n = 30). Zebrafish in cohort I received the intraperitoneal injection of PBS (10 μL). Zebrafish in cohort II received the microinjection of 250 CML μg suspended in 10 μL of PBS. Zebrafish in cohorts III, IV, V and VI received the microinjection of 250 μg CML suspended in 10 μL of each rHDL-(1:0), rHDL-(1:0.1), rHDL-(1:0.5), and rHDL-(1:1), respectively. Microinjection across all the cohorts was conducted after anaesthetizing zebrafish with 0.1% 2-phenoxyethanol.

The swimming behavior across all the cohorts was examined at 30 min and 60 min post-treatment by following previously outlined parameters [58]. The primary criteria for evaluating swimming activity encompassed tail fin motion and the occurrence of body convulsions [5,6,57]. The survivability of the zebrafish was examined after 3 h post-treatment. 

Three hours post treatment, the zebrafish across all the groups were sacrificed, and blood was collected immediately in a tube containing ethylenediaminetetraacetic acid (1 mM EDTA). Subsequently, the blood was processed for the quantification of aspartate transaminase (AST) and alanine transaminase (ALT) using the commercial kits (Asan Pharmaceutical, Hwasung, Republic of Korea), following the manufacturer’s instructions.

### 4.14. Hepatic Histology and Immunohistochemistry

The liver was surgically removed from different groups and preserved in 10% formalin for 24 h. After undergoing alcohol dehydration, the tissue was then fixed in paraffin. Subsequently, five μm thick sections were treated with poly-L-lysine and stained with hematoxylin and eosin (H&E). Examination of the stained tissue sections for morphological changes was conducted using an optical microscope (Motic Microscopy PA53MET, Hong Kong, China). The neutrophils in the different H&E-stained areas were quantified by microscopic examination across various groups. The neutrophil count was assessed in three distinct sections (n = 3) for each group, and the results are expressed as the percentage of neutrophil count in different groups relative to the 100% neutrophil count in the CML-injected group.

IL-6 production in hepatic tissue was quantified using immunohistochemical staining, following the established method [6]. The 5 μm thick tissue section was treated with a primary anti-IL-6 antibody (ab9324, Abcam, London, UK). After overnight incubation at 4 °C, the tissue section was processed using Envision + system Kits (code 40001, Dako, Denmark), which included horseradish peroxidase (HRP) conjugated-secondary antibody specific to the primary anti-IL-6 antibody. The quantification of Il-6 was conducted using Image J software version 1.53r (http://rsb.info.nih.gov/ij/, accessed on 18 July 2023). This involved converting the IL-6-stained area to an RGB stack and applying threshold levels between 20 (lower limit) and 100 (upper limit). This was done to enhance the visualization and to reduce the inclusion of background staining. All the images were processed using the same threshold values, and the resulting percentage area (corresponding to the IL-6-stained area) was determined.

### 4.15. Statistical Evaluation

The data is presented as mean ± SEM for each set of experiments conducted three times with duplicate samples. Group comparisons and statistical distinctions were assessed using a one-way analysis of variance (ANOVA) via SPSS software (version 29.0, SPSS, Inc., Chicago, IL, USA), followed by Tukey’s multiple range test. Spearman correlation analysis was employed to identify positive correlation between improved embryo survivability and enhanced CIGB-258 content in rHDL.

## 5. Conclusions

The incorporation of CIGB-258 improved the structural stability of apoA-I and rHDL particles and enhanced the beneficial functionality of HDL. CIGB-258 could bind well to phospholipid and cholesterol to stabilize apoA-I in the rHDL structure to form a larger particle size. The rHDL containing CIGB-258 enhanced the in vitro antioxidant ability against LDL oxidation, anti-glycation activity to protect HDL, and in vivo anti-inflammatory activity against CML toxicity in zebrafish adults and embryos. Overall, incorporating apoA-I and CIGB-258 in rHDL resulted in synergistic interactions that enhance the structural and functional correlations of HDL in a dose-dependent manner.

## Figures and Tables

**Table 1:** Change in the median WMF during isothermal denaturation by the urea treatment.

Name	Molar Ratio POPC:FC:apoA-I:CIGB-258	Median WMF (nm) ^1^	D_1/2_ ^2^ Urea, (M)	*r*	*p*
Lipid-free apoA-I	-	350.3	2.95	0.977	<0.001
rHDL-0	95:5:1:0	347.1	3.07	0.974	<0.001
rHDL-0.1	95:5:1:0.1	345.2	3.42	0.984	<0.001
rHDL-0.5	95:5:1:0.5	344.7	3.58	0.987	<0.001
rHDL-1	95:5:1:1	343.8	3.63	0.991	<0.001

^1^ WMF of apoA-I at median concentration of urea treatment (final 3.5 M). ^2^ D_1/2_ is a required urea concentration to reach 50% of denaturation of apoA-I by regression analysis. ApoA-I, apolipoprotein A-I (MW = 28,100); CIGB-258, Jusvinza^®^, (MW = 2987); FC, POPC, WMF, and MW denote free cholesterol (MW = 387), palmitoyloleoyl phosphatidylcholine (MW = 760), wavelength maximum fluorescence, and molecular weight, respectively.

**Table 2:** Characterization of rHDL containing apoA-I with different molar ratios of CIGB-258.

Name	Molar Ratio POPC:FC:apoA-I:CIGB-258 ^1^	Amount (μg) in 0.7 mL (POPC:FC:apoA-I:CIGB-258)	WMF ^2^ (nm)	Diameter ^3^ (nm)
ApoA-I	-	-	338.8 ± 0.1	-
CIGB-258	-	-	327.4 ± 0.1	-
rHDL-0	95:5:1:0	2560:67:1000:0	333.9 ± 0.1	60.2 ± 1.5
rHDL-0.1	95:5:1:0.1	2560:67:1000:10.6	333.1 ± 0.2	62.7 ± 2.3
rHDL-0.5	95:5:1:0.5	2560:67:1000:53	332.5 ± 0.0	80.3 ± 4.5
rHDL-1	95:5:1:1	2560:67:1000:106.6	332.1 ± 0.1	83.2 ± 3.4

^1^ The rHDLs were synthesized by sodium cholate dialysis method. ^2^ Wavelengths of maximum fluorescence (WMF) of the tryptophan (Trp) residues in apoA-I were determined from the uncorrected spectra using an FL6500 spectrofluorometer. ^3^ Particle size of each rHDL was determined by transmitted electron microscopy. ApoA-I, apolipoprotein A-I (MW = 28,100); CIGB-258, Jusvinza^®^, (MW = 2987); FC, free cholesterol (MW = 387); POPC, palmitoyloleoyl phosphatidylcholine (MW = 760); WMF, wavelength maximum fluorescence; MW, molecular weight. FC, free cholesterol.

## Data Availability

The data used to support the findings of this study are available from the corresponding author upon reasonable request.

## Supplementary Materials

The following supporting information can be downloaded at: https://www.mdpi.com/article/10.3390/ph17020165/s1, Video S1: Swimming pattern of CML + PBS group at 60 min post-injection. Video S2: Swimming pattern of CML + rHDL-(1:0) group at 60 min post-injection. Video S3: Swimming pattern of CML + rHDL-(1:0.1) group at 60 min post-injection. Video S4: Swimming pattern of CML + rHDL-(1:0.5) group at 60 min post-injection. Video S5: Swimming pattern of CML + rHDL-(1:1) group at 60 min post-injection.
